# Transcriptome Profiling of the Potato Exposed to French Marigold Essential Oil with a Special Emphasis on Leaf Starch Metabolism and Defense against Colorado Potato Beetle

**DOI:** 10.3390/plants10010172

**Published:** 2021-01-18

**Authors:** Sofija Stupar, Milan Dragićević, Vele Tešević, Jovana Stanković-Jeremić, Vuk Maksimović, Tatjana Ćosić, Nina Devrnja, Ljiljana Tubić, Aleksandar Cingel, Branka Vinterhalter, Slavica Ninković, Jelena Savić

**Affiliations:** 1 Institute for Biological Research “Siniša Stanković”—National Institute of Republic of Serbia, University of Belgrade, Bulevar despota Stefana 142, 11060 Belgrade, Serbia; sofija.stupar@ibiss.bg.ac.rs (S.S.); mdragicevic@ibiss.bg.ac.rs (M.D.); tatjana@ibiss.bg.ac.rs (T.Ć.); nina.devrnja@ibiss.bg.ac.rs (N.D.); tubic@ibiss.bg.ac.rs (L.T.); cingel@ibiss.bg.ac.rs (A.C.); horvat@ibiss.bg.ac.rs (B.V.); slavica@ibiss.bg.ac.rs (S.N.); 2 Faculty of Chemistry, University of Belgrade, Studentski trg 16, 11000 Belgrade, Serbia; vtesevic@chem.bg.ac.rs; 3 Institute of Chemistry, Technology and Metallurgy, Department of Chemistry, University of Belgrade, Njegoševa 12, 11000 Belgrade, Serbia; jovanas@chem.bg.ac.rs; 4 Institute for Multidisciplinary Research, University of Belgrade, Kneza Višeslava 1, 11030 Belgrade, Serbia; maxivuk@imsi.rs

**Keywords:** potato, French Marigold, essential oil, microarray, starch metabolism, Colorado potato beetle

## Abstract

Flower strips of French Marigold are commonly used pest repellents in potato fields. However, the effect of French Marigold volatiles on potato metabolism, physiology and induced defense is unknown. Thus, a microarray transcriptome analysis was performed to study the effects of French Marigold essential oil (EO) on laboratory-grown potato. After 8 h of exposure to EO, with gas chromatography/mass spectrometry (GC/MS)-detected terpinolene and limonene as dominant compounds, 2796 transcripts were differentially expressed with fold change >2 compared to expression in controls. A slightly higher number of transcripts had suppressed expression (1493 down- vs. 1303 up-regulated). Since transcripts, annotated to different photosynthesis-related processes, were mostly down-regulated, we selected a set of 10 genes involved in the leaf starch metabolism pathway, and validated microarray patterns using quantitative reverse transcription polymerase chain reaction (RT-qPCR). Except for decreased synthesis and induced decomposition of starch granule in leaves, 8 h long EO exposure slightly elevated the accumulation of sucrose compared to glucose and fructose in subjected potato plants. An in vitro feeding bioassay with Colorado potato beetle showed that EO-induced alternations on transcriptional level and in the sugars’ metabolism caused the enhancement of feeding behavior and overall development of the tested larvae. Results of comprehensive analysis of transcriptional responses in potato exposed to French Marigold EO provide a basis for further elucidation of molecular mechanisms underlying eco-physiological interactions in companion planting cropping systems.

## 1. Introduction

Dispersal of volatile organic compounds (VOCs) in the air is considered to be “the aboveground language” plants use for communication with other organisms. Regardless of whether plants produce and emit volatile metabolites constitutively and/or in response to external stimuli, these unique blends contribute to different plants’ physiological processes or send informative signals to others. Complex VOC mixtures referred to as essential oils (EOs) constitute the scent of flowers and the flavor of fruits [1], attract pollinators and seeds dispensers [2,3], repel and intoxicate herbivores, decoy parasites that prey on herbivores or their eggs [4] or suppress the growth of competitor plants [5]. Many VOCs exhibit potent antimicrobial properties contributing to fighting phytopathogenic organisms [6,7]. Being aware of these interactions, farmers have for decades planted aromatic and flowering species rich in EOs next to cultivating crops. This crop protection strategy, named companion planting, is applied in traditional agriculture worldwide and is being commonly adopted in modern organic farming as well [reviewed in [8]].

Besides intensively studied effects of volatile blends on different animal species, belonging either to beneficial or detrimental groups, the role of these compounds in plant-to-plant interactions is still poorly understood. Among the first, Arimura et al. [9,10,11] reported intensive genes activation in lima bean (*Phaseolus lunatus*) leaves that were exposed to volatiles released from the neighboring leaves infested with spider mites (*Tetranychus urticae*), under laboratory conditions. Affected genes were identified as part of a wide range of pathways related to the responses to pathogenesis, wounding, ethylene biosynthesis, flavonoid biosynthesis, posttranscriptional modifications, translations, chaperones, secondary signaling messengers, membrane transports, protein/peptide degradations, and photosynthesis. These findings, together with similar ones following [12,13,14], have led to postulations that VOCs could convey the messages of upcoming danger. They affect the physiology of neighboring conspecific or interspecific receiver plants and stimulate them to prepare defense strategies in advance for potential pest attacks [15,16,17,18].

The majority of reports dealing with plant-to-plant communication rely on well-established models that comprise pest-infested plants emitting herbivore-induced plant volatiles (HIPVs), neighboring receiver plants and analyses of consequently activated defense responses. The fact is that HIPV blends have specific composition differing from released EOs qualitatively and in abundance of particular compounds, with the special task to warn the neighbors. As a defense response to wounding or herbivore damage, plants produce and emit directly from the wound site so-called green leafy volatiles (GLVs) [19], potent signals for the transient transcriptional boost of a plethora of defense-related genes in neighboring plants [9,10,20,21]. Induced accumulation of defense-related compounds prior to a real attack could give an inherent advantage to plants enhancing their responsiveness to biotic stressors. Kessler et al. [22] provided evidence that volatiles from clipped sagebrush (*Artemisia tridentata*) increased the production of proteinase inhibitors in nearby tobacco plants subsequently causing higher mortality rate of feeding young *Manduca sexta* caterpillars. Elevated tolerance of valued crops to devastating herbivorous pests could offer a sustainable alternative to conventional deleterious pesticidal chemicals.

However, the continuous exposure of growing crops to inescapable EOs of companion plant species in the field is frequently neglected. As we mentioned before, flowering and aromatic plants are usually planted near crops in order to protect them from pests, but is their volatiles’ influence on crop plants completely negligible? Have receiver plants “learned to ignore” ubiquitous volatile signals and to respond only to herbivore-, pathogen- or damage-induced signals? Maintaining a high level of defense is considered “expensive” to plant metabolism, affecting overall plant performance [23]. This is of special importance for crops grown as food or feed, since the alternation in metabolism could lead to reduction in overall yield and nutritive values.

In order to obtain the answers to some of those questions and initiate the elucidation of molecular mechanisms underlying “communication” between crops and EO emitting companion plants, we set-up a controlled laboratory experiment in which potato plants had been exposed to French Marigold EO. Potato (*Solanum tuberosum* L.) is one of the most important and widely grown agricultural crops, the starchy tubers of which are present in the daily nutrition of over 1 billion people [24]. Current field protection practice against its major pest, Colorado potato beetle (CPB; *Leptinotarsa decemlineata* L.), relies mainly on broad spectrum of chemical pesticides. On the other hand, French Marigold (*Tagetes patula* L.) is one of the plants that can often be seen planted as flowers strips near potato in traditional and organic farming for its repellent effect on the CPB, the blister beetle and root-knot nematodes [25]. However, the influence that French Marigold EO could have on metabolism, defense mechanisms, pests’ resistance levels and yield of potato is still unknown.

A comprehensive cDNA microarray analysis of potato plants exposed to French Marigold EO for 8 h was performed to show overall transcriptome alternations induced by volatiles. An extensive bioinformatics survey was conducted in order to highlight the most affected metabolic pathways and particular genes, with a special focus placed on the leaf’s starch metabolism pathway. Expression profiles of selected potato genes related to this pathway were examined for the varied EO exposure periods. Finally, to analyze the potential of EO to induce defense of potato plants to Colorado potato beetle, an in vitro feeding bioassay was performed.

## 2. Results

### 2.1. French Marigold Essential Oil Composition

Essential oil from French Marigold was obtained by hydrodistillation with yield of 0.174% (*v*/*w*) as a pale yellow viscous oil with pungent odor. During GC/MS analysis, 42 different compounds were separated, of which 32 were identified (Table 1). These 32 compounds combined represented 97% of the total EO yield. The monoterpene fraction was dominant (70.0%), and the most abundant compounds were terpinolene (32.4%), limonene (14.7%), *E*-caryophyllene (12.2%), (*Z*)-*β*-ocimene (10.8%) and piperitone (8.2%).

### 2.2. Microarray

To confirm the ability of French Marigold EO volatiles to affect transcriptional processes in potato, we examined the gene expression profiles in potato plants exposed to EO for 8 h, using the Agilent custom set up full-genome microarray. Analysis revealed that 2796 transcripts had more than 2 times changed expression (fold change (FC) > 2) compared to expression levels in the control, making 7.38% of all 37891 potato sequences used for designing the probes, and 9.55% of 29260 probes used in differentially expressed (DE) analysis (Table 2).

A slightly higher number of analyzed transcripts had suppressed expression (1493 down-regulated vs. 1303 up-regulated transcripts). After performing more strict statistical cut-off, the number of affected transcripts had decreased (Table 2) revealing that 485 transcripts had FC > 5, 181 transcript FC > 10 and 8 transcripts even FC > 100.

### 2.3. Functional Annotation and Classification of Differentially Expressed (DE) Transcripts

**Figure 1:**
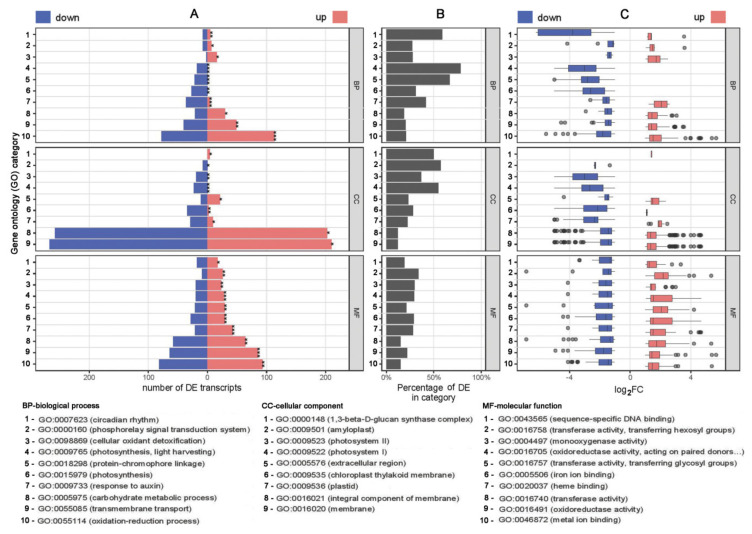
Annotations of the acknowledged DE transcripts to Gene Ontology (GO) categories: BP—biological process, CC—cellular component and MF—molecular function. Top 10 categories for BP and MF, and top 9 categories for CC, per the total number of DE transcripts, with significant over representation, are presented. (**A**) Number of down- (blue) and up -regulated (red) DE transcripts in each category. Category over representation *p*-values are indicated with asterisks: ***—*p*-value < 10^− 10^; **—*p*-value < 10^−5^; *—*p*-value < 10^−3^. (**B**) Percentage of DE transcripts from the total number of transcripts in the annotated category. (**C**) Boxplots of transcript log_2_FC values for down- (blue) and up -regulated (red) DE transcripts in the corresponding category.

**Figure 2:**
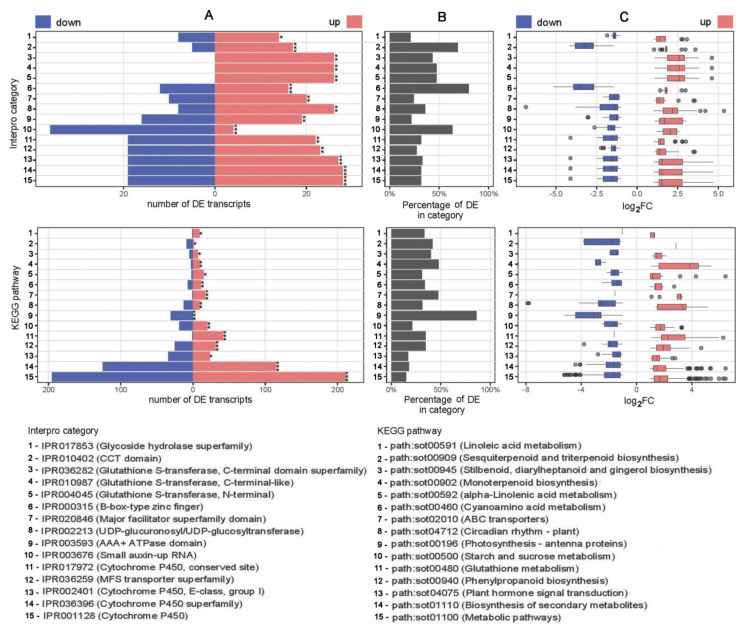
Annotations of the acknowledged DE transcripts to Interpro and Kyoto Encyclopedia of Genes and Genomes (KEGG) pathways associations. Top 15 categories per the total number of DE transcripts, with significant over representation are presented. (**A**) Number of down- (blue) and up -regulated (red) DE transcripts in each category. Category over representation *p*-values are indicated with asterisks: ***—*p*-value <10^−10^; **—*p*-value <10^−5^; *—*p*-value <10^−3^. (**B**) Percentage of DE transcripts from the total number of transcripts in the annotated category. (**C**) Boxplots of transcript log_2_FC values for down- (blue) and up -regulated (red) DE transcripts in the corresponding category.

The acknowledged DE transcripts were associated to Gene Ontology (GO), Interpro, and Kyoto Encyclopedia of Genes and Genomes (KEGG) annotations with gene ID by interfacing with Ensembl Plants database (Figure 1 and Figure 2; Supplementary Table S2).

GO assignments classified the predicted functions of 2796 transcripts into three main categories: BP—biological process, CC—cellular component and MF—molecular function (Figure 1; Supplementary Table S2). The majority of DE transcripts in CC category were coding for components of the membranes (GO:0016020; 477 transcripts), with almost equal distribution of up- and down-regulated ones. Noticeably, all DE transcripts belonging to Photosystems I and II (GO:0009522 and GO:0009523 with 23 and 19 DE transcripts, respectively), and amyloplasts (GO:0009501 with 8 DE transcripts) were down-regulated by the effect of EO. Other categories related to plastids (GO:0009536) and chloroplast thylakoid membranes (GO:0009535) were down-regulated in the majority. In concordance, the highest number of transcripts within BP category were related to transmembrane transport (GO:0055085 with 89 transcripts) and oxido-reduction processes (GO:0055114 with 191 transcripts), whereas transcripts with roles in photosynthetic processes (light harvesting GO:0009765, protein-chromophore linkage GO:0018298 and photosynthesis GO:0015979) were again all down-regulated in EO-exposed potato.

Interpro, used for identification of conserved domains or functional units within the protein query sequences, revealed that 1706 DE transcripts had Interpro annotation, while 551 DE transcripts were assigned to 111 significantly over represented (*p*-value < 10^−3^) Interpro categories (Supplementary Table S2). Statistical analysis showed that cytochrome P450 related categories (IPR001128, IPR036396 and IPR002401) were the most prominent families, but the most affected were B-box-type zinc finger and CCT domains with DE transcripts constituting 80% (28/35) and 68.75% (22/32) of the total annotated transcripts in each respective category. Again, among 15 most affected categories are glutathione S-transferase C- and N-terminal domains (IPR036282, IPR010987 and IPR004045) within whom all DE transcripts were up-regulated by EO treatment (Figure 2).

In the KEGG database, 671 DE transcripts were annotated into 112 pathways, with 15 pathways (554 DE transcripts) significantly over represented (*p*-value < 10^−3^) in EO exposed plants (Supplementary Table S2). “Metabolic pathways” (sot01100) with 406 DE transcripts was the most represented group and was followed by “biosynthesis of secondary metabolites” (sot01110, 240 DE transcripts). Among the most affected pathways were those related to “Glutathione metabolism”. From 124 identified transcripts belonging to this pathway 43 were with altered expression compared to controls, and 42 of them were significantly up-regulated. By contrast, all significantly affected genes (31 DE transcripts) from the “Photosynthesis-antenna proteins” pathway (sot00196) were down-regulated (Figure 2).

### 2.4. Effect of French Marigold Essential Oil on Expression of Genes Involved in Potato Leaf Starch Metabolism

Biostatistical analysis of microarray-obtained data revealed 39 sequences with roles clearly annotated to starch metabolism in chloroplasts (Table 3).

**Figure 3:**
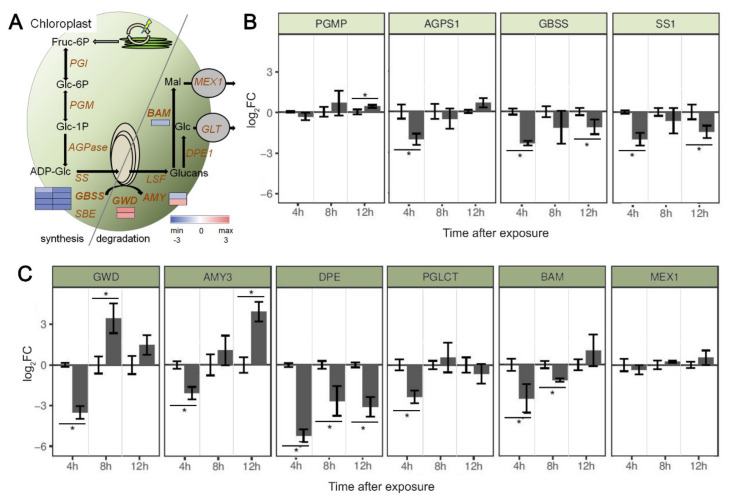
(**A**) Proposed starch metabolism pathway and associated gene expression in potato leaves exposed to French Marigold EO for 8 h. Expression patterns of DE transcripts (*p* ≤ 0.05) obtained by cDNA microarray analysis were presented as a single rectangle on color-coded heat maps (blue, down-regulated; red, up-regulated). Fruc-6P, Fructose-6-phosphate; PGI, phosphoglucoisomerase; Glc-6P, glucose 6-phosphate; Glc-1P, glucose 1-phosphate; PGM, phosphoglucomutase; AGPase, ADP-glucose pyrophosphorylase; ADP-Glc, ADP-glucose; SS, starch synthase; GBSS, granule-bound starch synthase; SBE, starch branching enzyme; GWD, glucan, water dikinase; AMY, α-amylase; LSF, Like starch-excess Four; BAM, β-amylase; DPE, disproportionating enzyme; GLT, glucose transporter; MEX1, maltose transporter; Mal, maltose; Glc, glucose. RT-qPCR obtained expression profiles of potato leaf genes involved in starch (**B**) biosynthesis and (**C**) degradation after exposure to French Marigold EO for different time periods (4, 8 and 12 h). The fold change (FC) of the genes expression were obtained after ΔΔCt normalization to the expression of reference 18S gene and to the expression in non-exposed controls (left bars; log_2_FC = 0) for each time point, and presented after log_2_ transformation. For each gene at each time point, Welch’s *t*-test was used, and the obtained *p*-values were jointly adjusted using the FDR method for multiple comparisons. Asterisks denote adjusted *p* ≤ 5 × 10^−2^ (n = 3).

Expression profiles of genes involved in starch metabolism from potato leaf samples exposed to French Marigold EO for different period of time were evaluated by quantitative reverse transcription polymerase chain reaction (RT-qPCR, Figure 3). Selected genes included ones coding for enzymes directly involved in starch synthesis based on literature, granule-bound starch synthase (*GBSS*; XM_015307086.1) and starch synthase (*SS1*; NM_001288145.1), as well as for two upstream enzymes phosphoglucomutase (*PGMP*; NM_001288352.1) and glucose-1-phosphate adenylyltransferase (*AGPS1*; XM_006365058.2), responsible for fructose/glucose conversion into substrates for starch synthesis. Enzymes involved in starch hydrolytic degradation—glucan water dikinase (*GWD*; NM_001288123.1), disproportionating enzyme (*DPE*; NM_001287852.1), α-amylase (*AMY3*; XM_006357203.2) and β-amylase (*BAM*; XM_006340834.2) were also analyzed. In addition, enzymes coding for downstream transmembrane transporters for glucose (*PGLCT*; XM_015313620.1) and maltose (*MEX1*; XM_006356232.2) were also in focus.

In general, expression patterns of genes involved in starch metabolism obtained by RT-qPCR (Figure 3B,C) were comparable to expression patterns obtained by microarray (Figure 3A), with general down-regulation of genes involved in starch synthesis and up-regulation of the first steps involved in starch degradation, followed by decreased activity of genes coding for downstream degradation.

The expression patterns obtained for genes involved in starch synthesis showed no significant differences in comparison to the control plants after 8 h long exposure to EO for all tested genes (Figure 3B). However, significant down-regulation was recorded for 3 of 4 analyzed genes (*AGPS1*, *GBSS* and *SS1*) when plants were exposed for the shorter period. Strong deactivation of genes directly involved in starch granule formation (*GBSS* and *SS1*) was also observed after prolonged exposure to EO of up to 12 h.

*GWD* and *AMY3* genes showed significant differences in expression levels after exposure to EO in comparison with non-exposed controls. While short-term exposure (4 h) had a strong down-regulating effect, after prolonged exposure these two genes had elevated transcription activity with up to 3.4 and 4.2 log_2_FC for *GWD* after 8 h and *AMY3* after 12 h, respectively. The other two genes responsible for starch decomposition, *DPE* and *BAM*, expressed the opposite pattern of expression, with strong reduction in transcript accumulation after exposure to EO. *PGLCT* and *MEX1* expressions were unchanged, except for *PGLCT* after 4 h.

### 2.5. Soluble Sugars Content

The content of soluble monosaccharides, fructose and glucose, generally decreased in potato plants exposed to EO for 8 h, in respect to control plants (Table 4). The higher alternation was recorded for glucose, concentration of which was almost halved. By contrast, accumulation of disaccharide sucrose was increased after EO exposure. However, all recorded changes in sugars concentrations were not statistically significant according to Student’s *t*-test.

### 2.6. Growth and Development of Colorado Potato Beetle Feeding on Essential Oil (EO)-Exposed Potato

**Figure 4:**
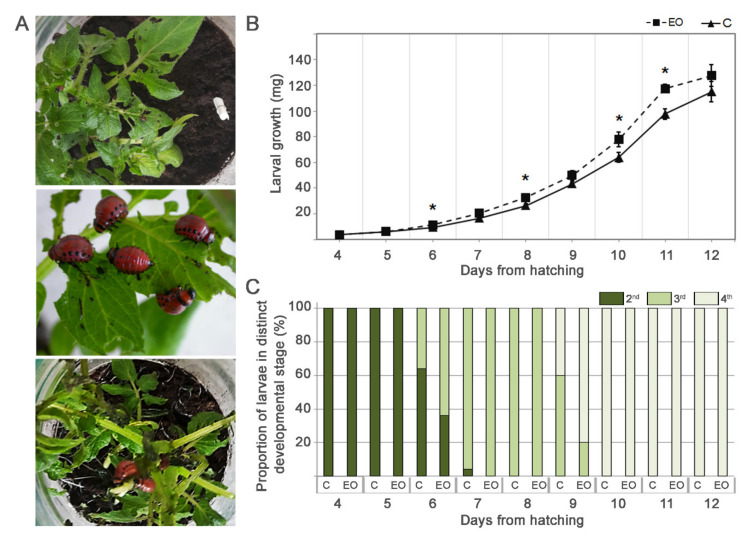
Effect of French Marigold EO exposure of potato plants on feeding Colorado potato beetles (CPB) growth and developmental dynamics. (**A**) Feeding of CPB larvae from 2nd instar placed on potato plants previously exposed to EO for 8 h, throughout 3rd and 4th instar larvae on defoliated potato plants after 8 days of feeding. (**B**) Larval growth was assessed through daily weight measures for larvae fed on EO-exposed (EO) and control (C) potato plants. (**C**) Duration of 2nd through 4th larval stages. All values are expressed as mean ± SE (n = 25). Means denoted with * are significantly different according to the least significant difference (LSD) test (*p* ≤ 5 × 10^−2^), within each time point.

Feeding on potato plants previously exposed to French Marigold EO for 8 h (Figure 4A), altered CPB larval growth (Figure 4B) and dynamic of development (Figure 4C). During the first 2 days of feeding, larvae of the 2nd instar gained weight with the same dynamic on exposed and control plants. The first significant differences were observed at the third day of feeding (and 7th day of hatching), at which larvae from EO exposed plants began to gain weight more intensively than controls (11.36 mg vs. 9.52 mg, respectively; F = 5.757, *p* = 0.043), and pass to the next developmental stage faster (64% vs. 36% in 3rd stage for EO-exposed vs. controls). These differences became even more pronounced as feeding continued. Thus, during the entire larval developmental process larvae fed and grew faster, and molted earlier on EO-exposed leaves than larvae reared on control ones. Maximum weight gains at the late 4th stage and decreased feeding at the onset of pupation were observed earlier in larvae fed on the plants exposed to EO.

However, at the end of larval development, all measured larvae, from both experimental groups (Figure 4C), entered into the prepupal stage with similar weights (127.53 mg vs. 115.00 mg for EO-exposed and controls, respectively; F = 1.159, *p* = 0.313). In addition, survival rates were 100% for all, and no significant effect of EO-exposed plants consumption on pupation and adult emergence was observed (data not shown).

## 3. Discussion

In order to investigate whether volatiles from French Marigold affect physiology and prime defense of interspecific plants, we set up the controlled laboratory experiment in which potato plants were exposed to French Marigold EO.

As with other plant species, potato has the ability to “sense” VOCs and efficiently adapt to current environmental factors and biotic interactions. Although it was hypothesized in the past that plants possess specific protein receptor-mediated recognition system for perceiving VOC cues, no such receptors for volatiles other than ethylene have been identified [26]. However, observed significant transcriptional response in potato plants exposed to French Marigold EO, with 1493 down-regulated and 1303 up-regulated transcripts, proves the efficient uptake of volatiles and their influence on gene expression. In our experiments, potato plants were exposed to the EO during the daylight regime, since it has been generally observed that uptake of VOCs is largely conducted through the stomata under conditions of illumination when they are open [27]. This process is conducted due to persistence of a physicochemical gradient from the ambient air to the leaves, and stomata act as a chemical sink [28].

In the EO isolated from flowering French Marigold, the monoterpene fraction of volatile compounds was dominant (70% of total EO), with terpinolene and limonene as the most abundant ones. It is still not clear how these highly hydrophobic molecules pass through cell membranes and enter the cytosol. In general, volatiles taken up by a plant frequently undergo glycosylation and glutathionylation, resulting in their conversion to non-volatile compounds with physiological functions [29]. While glycosylation of volatiles is well described, with identified different UDP-glycosyltransferases as key factors [30], the role of glutathionylation in converting the VOCs from gaseous to liquid phase is poorly investigated. Interestingly, one of the most striking groups of transcripts that had an unambiguous pattern of expression was annotated as glutathione S-transferases (GSTS). Of the 60 analyzed transcripts belonging to this category (IPR036282), 26 of them were differentially expressed with FC >2, and all of them were up-regulated by EO treatment. Involvement of cytosolic GSTS in formation of conjugates with glutathione (GSH) during detoxifying xenobiotics other than VOCs is elaborated for numerous plant species [31,32,33]. It is considered that generated GSH conjugates are mostly catabolized and transported to the vacuole, which leads to plant resistance and adaptation to a variety of physical and chemical stresses encountered in the environment. Moreover, Mhamadi et al. [34] showed that changes in total GSH level and GSH redox potential influenced jasmonic and salicylic acids signaling, both involved in stress response. The gluthatione-based response mechanism certainly is an important component of observed VOC-induced responses in the potato, and will be in focus of our subsequent investigation.

On the other hand, it is evident that received VOCs could trigger many plant processes before being scavenged [35]. Thus, the effects of exogenous EOs or solely monoterpenes on chlorophyll concentrations and overall photosynthetic efficiency of acceptor plants have been frequently demonstrated [36,37]. The negative impact of limonene spraying on chlorophyll fluorescence, net photosynthesis and stomatal function was presented in two cabbage and two carrot cultivars [38]. Similarly, in potato exposed to French Marigold EO, transcripts defined as being involved in photosynthetic processes (GO:0009765, light harvesting; GO:0018298, protein-chromophore linkage; GO:0015979, photosynthesis), were generally down-regulated compared to control. Since potato is grown worldwide for its starchy tubers used as food or feed, any disturbance in photosynthesis rates and related starch metabolism could affect the production efficiency and lead to an undesirable decrease of tuber yield.

Analysis of expression dynamic of genes involved in potato leaf starch metabolism revealed a decrease of both starch synthesis and degradation in plants exposed to French Marigold EO for 4 h, but induced only degradation if exposure lasted for a longer period of time (8 and 12 h). Genes coding for enzymes responsible for transformation of Fru-6P (the first metabolite entering into the starch biosynthesis pathway from the Calvin cycle) into building blocks for starch synthesis, plastidial phosphoglucomutase (*PGMP*) and ADP-glucose pyrophosphorylase (*AGPase*), generally had similar expression intensities in plants whether exposed to EO or not. However, genes coding for further steps and incorporation of ADP-Glc units into amylose and amylopectin chains, granule-bound starch synthase (*GBSS*) and starch synthase (*SS1*), showed a more intensive response to EO exposure with up to almost 5 times reduced expression (log_2_FC = −2.4). One of the possible explanations for observed down-regulation could be that in the VOC enriched atmosphere, intensive uptake of volatile compounds through stomata [39] influenced CO_2_ uptake. This disturbance might further lead to declined photosynthesis and reduced accumulation of Fru-6P.

Under normal physiological conditions, the so-called “primary or transitory starch” formed during the day in leaves, is decomposed during the night. The glucans obtained are further mobilized, supplying carbon for continued sucrose synthesis and sustaining the metabolism when photosynthesis is not possible [40]. However, our results showed that this process can be altered also during the day under the influence of some exogenous factors other than light and/or diurnal regulation. Thus, glucan water kinase (GWD) and alfa-amylase (AMY3), responsible for the breakdown of granular starch by phosphorylation, showed significant alternations in expression patterns in plants exposed to French Marigold EO. It is interesting that both genes showed a significant decrease of expression after 4 h of exposure to EO, while the prolonged influence of VOCs switched the mode of action and induced up to almost 20 times more intensive transcription (log_2_FC = 3.9). In addition, this down-regulation pattern in plants shortly exposed to EO persisted for all downstream genes coding for enzymes involved in subsequent glucans decomposition (*DPE*, responsible for conversion of malto-oligosacharides into Glu, and *BAM*, responsible for maltose release), and products export (*PGLCT* and *MEX1*, for glucose and maltose passage, respectively). According to the results presented, during the first hours of French Marigold EO evaporation starch metabolism in potato plants was completely affected and slowed down both synthesis and degradation steps. It is known that plants usually react more intensively to intense contiguous stimulus, by inducing defense capacities and investing all capacities into the accumulation of defense-related cues [41,42]. However, prolonged stimuli give a chance to the plant to respond in a more energy-effective way, maintaining the primary metabolism at the needful level [43,44]. Also it is possible that different evaporation dynamic of VOCs belonging to different classes could induce varied plant responses. The dominant group of compounds in French Marigold EO are monoterpens, which are the most volatile, but semi-volatile sesquiterpens are also present in this EO which we used to treat potato plants. In intact plants VOCs are accumulated and emitted as an ecophysiological response to different developmental and exogenous factors, but in our experimental set up, the EO was applied on filter papers and allowed to evaporate into jars where potato plants were grown. Thus, evaporation dynamic was the consequence of only physico-chemical properties of different classes of VOC present in EO [45].

Disturbed carbohydrate metabolism in leaves of potato plants exposed to French Marigold EO was reflected in changed accumulation of sugars. Although statistics did not show significant differences between experimental groups (control and EO-exposed plants), due to wide variability within biological replicates of each group, it was shown that exposed plants had less glucose and fructose, while the content of sucrose was increased. This metabolic alternation could be considered as “on purpose” turn to defense induction. Recently, the term “high-sugar resistance” was proposed by Ferri et al. [46] after comprehensive research in which the promotion of phenylpropanoid biosynthesis, involved in plant defense mechanisms, was detected upon increasing sucrose concentrations in grapevine cell cultures. The following investigations showed that, in this manner, sucrose acts predominantly as a signal molecule, inducing defense responses [47,48].

However, results of a CPB biotest showed no detrimental effects of French Marigold EO pretreatment of potato plants on this pest. In contrast to what was expected, larvae reared on potato plants which had been exposed to EO for 8 h before the start of the biotest, accumulated more biomass and molted earlier than those from control plants. It is well known that the feeding behaviors and overall development of the CPB are mostly attributed to the chemical composition of the potato foliage reviewed in [49]. Sucrose is among major nutrients responsible for stimulating feeding behavior in CPB larvae, while fructose and glucose were shown to have little effect on the development of the CPB [50]. Thus, CPB larvae in our experiment used sucrose-enriched potato leaves, to gain higher biomass and attain the critical mass necessary for the molting process faster. At the same time, relatively short (8 h), sole exposure of potato to French Marigold EO prior to insect feeding, was not sufficient for increasing the resistance to this pest. Although the EO induced the accumulation of some defense-related transcripts, for more intensive defense and elevated resistance, exposure to VOCs should be prolonged and/or repeated, allowing the plants to accumulate the defense-related agents in necessary quantities. Sukegawa et al. [51] reported strong enhancement in RNA levels for two defense-related genes, trypsin inhibitor (*TI*) and pathogenesis-related 1 (*PR1*) genes in soybean leaves exposed to mint plants grown nearby. Larvae of *Spodoptera litura* fed on receiver soybeans exhibited a lower weight gain (up to 40% compared to larvae grown on non-receiving soybean plants). Similarly, potato plants in which exposure to constitutively emitting onion plant volatiles, activated the indirect defense strategy and attracted the potato herbivores’ enemy *Coccinella septempunctata* [52]. On the other hand, the unintended effects of a short single exposure could even be favorable for pests under field conditions due to shorter exposure to predators and pathogens [53], as well as the faster spread of progeny, all as a result of the accelerated development process. However, it would be of great interest to analyze the prolonged effect of EO-induced sucrose accumulation on the plant immune system and subsequent consequences on feeding larvae, as one more aspect of the co-evolutionary “arms race” [54] between potato and CPB.

## 4. Materials and Methods

### 4.1. Essential Oil Isolation and Quantification

French Marigold (*Tagetes patula* L.) essential oil (EO) was isolated from dry above-ground parts of plants by hydrodistillation using a Clevenger-type apparatus. Plant material used for EO isolation was collected at the blooming stage from a private garden near Belgrade (Jajinci village, Serbia) and dried in a shaded place at room temperature. Isolated EO was kept in a dark glass bottle at a temperature of 4 °C. The total yield of collected EO was presented as a percentage (%) of obtained volume (mL) in total mass of dry plant material (g).

The gas chromatography (GC) and gas chromatography/mass spectrometry (GC-MS) analyses were performed on an Agilent 7890A GC system equipped with 5975C inert XL EI/CI MSD and a FID detector connected by capillary flow technology 2-way splitter with make-up gas. A DB-5 capillary column (30 m × 0.25 mm × 0.25 μm) was used. Samples were injected in split mode (10:1). The injection volume was 1 μL and the injector temperature was 220 °C. The carrier gas (He) flow rate was 1.0 mL min^−1^ at 210 °C (constant pressure mode). The column temperature was linearly programmed in a range of 60–240 °C at a rate of 3 °C min^−1^. The transfer line was heated at 240 °C. The FID detector temperature was 300 °C. EI mass spectra (70 eV) were obtained in the *m/z* range of 35–550 atomic mass units (amu). The ion source and quadrupole temperatures were 300 °C and 150 °C, respectively. A library search and mass spectral deconvolution and extraction were performed using NIST AMDIS (Automated Mass Spectral Deconvolution and Identification System) software version 2.64.113.71, using retention index (RI) calibration data analysis parameters with ‘strong’ level and 10% penalty for compounds without an RI. The retention indices were experimentally determined using the standard method involving retention times of n-alkanes, injected after the EO under the same chromatographic conditions.

### 4.2. Experimental Design

Potato plants (Désirée cultivar) used as EO receivers were germinated from tubers collected from a chemically untreated private field (Sremska Mitrovica, Serbia). Sprouted tubers were planted in soil mixtures (60% peat, 20% humus, 20% sand, *v*/*v*/*v*; Bašta d.o.o., Zrenjanin, Serbia) directly in 5 L glass jars (6 plants per treatment) and grown under standardized conditions (16 h light/8 h dark interval, with 70 µmol m^−2^s^−1^ flux provided by led lights at 22 ± 1 °C) with regular watering. After 18 days, fully expanded potato plants (receiver plants) were exposed to French Marigold EO for 4, 8 and 12 h. Essential oil (10 µL) was applied on filter papers (Whatman 3, 1 × 1 cm), that were placed in the jar on the metal holder, avoiding contact with plant parts and allowing EO to evaporate. In the jars containing control plants for each time point, no EO was added and material was collected at the same time points as for exposed ones (4, 8 or 12 h after exposing the receiver plants to EO). Jars with exposed and control plants were tightly closed with lids and sealed with parafilm.

Three or four (out of 6) representative receiver plants, exposed to EO for either 4 h, 8 h or 12 h and control plants for each time point, were removed from the jars and the leaves were collected and grounded in liquid nitrogen instantly. Powdered material was separated for further analyses (RNA isolation and soluble sugar content measurements). For microarray analysis plants exposed to EO for 8 h, and corresponding control plants, were selected.

For insect feeding bioassay, potato plants were grown in the same way. Five plants, each in separate jars, were used for both groups, i.e., plants exposed to EO for 8 h prior to CPB larvae feeding and control plants.

### 4.3. RNA Isolation

RNA isolation from 100 mg of powdered potato leaf tissue was performed according to a slightly modified Gasic et al. [55] protocol [56]. Briefly, total RNA were extracted with ice cold 0.1 M TRIS-HCl pH 8 buffer with 2% (*w*/*v*) cetyl trimethylammonium bromide (CTAB), 2% (*w*/*v*) polyvinylpyrrolidone (PVP), 25 mM ethylenediaminetetraacetic acid (EDTA), 2 M NaCl, 0.5 g L^−1^ spermidine and 2% (*v*/*v*) β-mercaptoethanol. RNA molecules were separated with subsequent 7.5 M LiCl and 3 M sodium acetate pH 5.5 precipitation after two series of chloroform:isoamylalcohol (24:1, *v*/*v*) purification. Concentration and purity of aqueous RNA extracts were evaluated by NanoPhotometer^®^ N60 (Implen GmbH, München, Germany). For microarray analysis RNA quality check was additionally performed on Agilent 2100 Bioanalyzer (Agilent Technologies, Palo Alto, CA, USA). The extracted RNA was used for microarray analysis and for RT-qPCR.

### 4.4. Microarray Analysis

Microarray analysis was performed using de novo assembled Agilent^®^ SurePrint G3 CustomPotato GE 8 × 60 K Array platform. For 37891 available potato sequences, total of 51014 probes were designed. For microarray gene expression analysis 100 ng of total RNA from each sample was linearly amplified and labeled with Cy3-dCTP using Agilent’s Quick Amp Labeling Kit according to the Agilent One-Color Microarray-Based Gene Expression Analysis protocol (Agilent Technologies, Inc., Santa Clara, CA, USA, V 6.5, 2010). The labeled cRNAs were purified by RNAeasy Mini Kit (Qiagen, Germantown, MD, USA), and the concentration and specific activity of the labeled cRNAs (pmol Cy3 μg^−1^ cRNA) were measured by NanoDrop ND-1000 spectrophotometer (Thermo Fisher Scientific, Wilmington, DE, USA). For each labeled cRNA, 600 ng was fragmented by adding 5 μL 10 × blocking agent and 1 μL of 25 × fragmentation buffer, and then heated at 60 °C for 30 min. Finally, 25 μL 2 × GE hybridization buffer was added to dilute the labeled cRNA. For the 8 × 60 K chip format 40 μL of hybridization solution was dispensed into the gasket slide. The slides were incubated for 17 h at 65 °C in an Agilent hybridization oven then washed at room temperature by using the Agilent One-Color Microarray-Based Gene Expression Analysis protocol (Agilent Technologies, V 6.5, 2010). The hybridized array was immediately scanned with an Agilent Microarray Scanner D (Agilent Technologies, Inc., Santa Clara, CA, USA).

DNA microarray data processing was performed in R 3.6 (R Core Team, 2019) using the package limma 3.40.6 [57]. Background correction was performed using maximum likelihood estimation for the normal-exponential convolution model [58]. Between array normalization was performed using ordinary cyclic loess normalization applied to all possible pairs of arrays [59]. Afterwards, the expression data was log_2_ transformed. Probes which had low foreground compared to background, as reported by the Agilent Feature Extraction Software (v11.0.1.1), in 5 or more arrays were discarded and the signal from the remaining probes was averaged per probe. In order to analyze data on the level of transcripts we examined the variance of the probe signals and kept the maximum variance probe per each transcript across arrays.

Differential expression was performed by fitting a linear model for each transcript. Moderated *t*-statistics, moderated F-statistic, and log-odds of differential expression were estimated by robust empirical Bayes moderation using the limma-trend method [60]. Adjustment of the degree of the false positive rate (*p*-value) was performed using the Benjamini and Hochberg [61] FDR method. Two criterions were used to label differentially expressed (DE) transcripts—FDR adjusted *p*-value < 5×10^−2^ and the minimum absolute log2 fold change (FC) required was set to 1 (FC of 2 between control and treatment).

The R package biomartr [62] was used to associate GO, KEGG and Interpro annotations with gene ID by interfacing with Ensembl Plants (http://plants.ensembl.org, accessed October 2019) database. Selection-unbiased testing for category enrichment amongst DE transcripts was performed using the goseq [63] R package. To obtain unbiased category enrichment scores, in regards to transcript lengths, Wallenius non-central hypergeometric distribution [64] was used to calculate the *p*-values. These *p*-values have not been corrected for multiple hypothesis testing since annotation terms used here such as GO terms, KEGG pathways and Interpro accessions are often overlapping, so standard methods of *p*-value adjustment may be very conservative.

### 4.5. Quantitative Reverse Transcription Polymerase Chain Reaction (RT-qPCR) Analysis

In order to analyze expression dynamics of genes included in starch metabolism of EO-exposed potato RT-qPCR analysis was conducted. The expression levels were estimated in samples collected from plants exposed to EO for 4 h, 8 h and 12 h. Remaining of genomic DNA from isolated total RNA were removed by treatment of 1 µg of RNA with DNase I (Thermo Scientific, Waltham, MA, USA) for 30 min at 37 °C. Subsequently, GeneAmp^®^ RNA PCR Core Kit (Perkin Elmer Life Sciences, Boston, MA, USA) was used for reverse transcription (RT) performed at 37 °C for 30 min and 95 °C for 5 min.

qPCR was performed on the Applied Biosystems QuantStudio 3 Real-Time PCR System using SYBR Green I (Maxima SYBR Green/ROX Kit, Thermo Scientific, Waltham, MA, USA) in biological triplicates. Reaction mixture contained 5 µL of SYBR Master Mix, 0.6 µL (corresponding to 0.3 µM) of each primer (forward and reverse) and 1 µL of cDNA (corresponding to 20 ng). Primers for selected genes involved in potato leaf starch metabolism (*PGMP*, *AGPS1*, *GBSS*, *SS1*, *GWD*, *AMY3*, *DPE*, *PGLCT*, *BAM*, *MEX1*; Supplementary Table S1) were designed using Primer-BLAST (www.ncbi.nlm.nih.gov/tools/primer-blast) [65]. Specificity of all primers was validated by BLAST, RT-PCR products separation on 1.2% agarose gels and analysis of melting curves during qPCR amplification. qPCR reaction conditions included initial denaturation at 95 °C for 5 min, followed by 40 cycles of denaturation at 95 °C for 30 s, annealing at 60 °C for 1 min, and extension at 72 °C for 1 min, with final extension at 72 °C for 10 min. Additional melting curve analyses were performed by cooling the reactions to 60 °C and then increasing the temperature to 95 °C with a slope of 0.1 °C s^−1^, while measuring the fluorescence continuously. The results were analyzed using QuantStudio™ Design and Analysis Software version 1.4 (Thermo Fisher Scientific, Waltham, MA, USA). The expression levels of tested genes were normalized to the internal control 18S rRNA gene (Supplementary Table S1) expression levels, and calculated relative to the non-exposed control according to the ΔΔCt method [66]. The results were presented as log_2_ transformation of fold changes (log_2_FC).

In order to calculate the efficiency of amplification process for each analyzed gene, the standard curves were constructed using 10 × serial dilutions (from 10^8^ to 10^2^ copies per μL). Standards were made from cDNA fragments which were amplified by PCR using primers from Supplementary Table S1. Amplicons were isolated from the agarose gel by GeneJET Gel Extraction Kit (Thermo Scientific, Waltham, MA, USA) and quantified by ND-1000 Spectrophotometer (NanoDrop). Formula from the link http://endmemo.com/bio/dnacopynum.php was used for making the standards using concentration of the isolates and amplicon length. Efficiency (E) was calculated from the slopes using the E = 10^(−1/Ct slope)^ formula.

Gene expression data were statistically processed in R. For each gene at each time point Welch’s *t*-test was used to estimate ΔΔCt differences between EO-exposed and control groups. Obtained *p*-values were adjusted using the Benjamini and Hochberg [61] FDR method.

### 4.6. Analysis of Soluble Sugars

Powdered potato leaves (100 mg), collected from plants exposed to Marigold EO for 8 h (or non-exposed for controls), were subjected to extraction with 1 mL of 80% methanol. After 10 s of sample vortexing, further extraction in an ultrasonic bath was performed for 10 min. The extracts were centrifuged for 10 min at 14,000× *g* and supernatants were filtered through a syringe filter with pore size 0.2 μm (Econofilter, Agilent Technologies Inc., Santa Clara, CA, USA) prior to analysis.

High-performance liquid chromatography with pulsed amperometric detector (HPLC-PAD) analysis was performed on Waters chromatographic system consisted of 1525 binary pumps, 1510 thermostated column compartment, and 2465 electrochemical detector (Waters, Milford, MA, USA), equipped with a 3 mm gold working and hydrogen reference electrode. Rheodyne 7725i (Rheodyne, Rohnert Park, CA, USA) manual injector was used and injections were performed with 25 µL SGE precise syringes (SGE Inc., Pflugerville, TX, USA). Sugar separation was performed on CarboPac PA1 (Dionex, Sunnyvale, CA, USA) 250 × 4 mm column equipped with a corresponding CarboPac PA1 guard column, using 100 mM NaOH (low carbonate, J.T. Baker, Deventer, Holland) as eluent. Sugars were eluted for 20 min at a flow rate of 1 mL min^−1^ at a constant temperature of 30 °C. Signals were detected in pulse mode with the following waveforms: E1 = +0.15 V for 300 ms; E2 = +0.75 V for 150 ms; E3 = −0.80 V for 200 ms, and with 100 ms of integration time. Data response setting was from 0–1000 mV, with a sampling rate of 0.1 mV s^−1^, filter timescale of 0.2 s, and sensitivity set from 2 to 5 µA for the full mV scale. Quantification was performed by the external standard method, using pure standard compounds as references for the concentration and retention time, respectively. Data acquisition and evaluation were carried out by Waters Empower 2 software (Waters, Milford, MA, USA).

All data were statistically processed in StatSoft Inc. STATISTICA, version 7 (2004) and subjected to Student’s *t*-test. Results were presented by means of three separate biological replicates ± standard errors (SE).

### 4.7. Colorado Potato Beetle Feeding Bioassay

Fresh potato Désirée leaves with egg clusters of Colorado potato beetle (*Leptinotarsa decemlineata* L.) were collected from pesticide-untreated fields (Šimanovci, Belgrade nearby, Serbia) during the summer. Detached leaves were placed in plastic Petri dishes (90 mm in diameter) on moist filter paper and incubated at 24 ± 1 °C. After larvae started to hatch, fresh potato leaves were added daily. Four days after hatching, larvae entering the 2nd instar (L2) were randomly selected from different clusters and uniformly distributed on potato plants previously exposed to French Marigold EO for 8 h, as well as onto non-exposed control plants (5 larvae per plant). Larvae were fed for the next 9 days, after which they slowed down upon feeding and entered the prepupal stage. The larval weights and molting events of individual larvae were measured and monitored daily to determine biomass accumulation and duration of each instar (2nd, 3rd and 4th).

Data from larval weight measurements were subjected to one-way analysis of variance (ANOVA) in StatSoft Inc. STATISTICA and results for each time point (4 h, 8 h and 12 h) were presented as means ± standard errors (SE) for n = 25. Differences between the corresponding means were compared using the post hoc least significant difference (LSD) test at *p* ≤ 5 × 10^−2^.

## 5. Conclusions

Results of comprehensive analysis of transcriptional responses in potato plants exposed to French Marigold EO will help in better understanding the eco-physiological interactions in companion planting cropping systems. By a so far unrevealed mechanism, volatiles from French Marigold EO were taken up into potato leaves and induced significant transcriptional alterations. Except for decreased synthesis and induced decomposition of starch granule in leaves, 8 h long EO exposure slightly elevated accumulation of sucrose compared to glucose and fructose in the subjected potato plants. The higher content of sucrose gave better nutritional quality to the potato leaves and unintentionally enabled the enhancement of feeding behavior and overall development of the CPB. According to transcriptional patterns obtained, we conclude that single-shot exposure to French Marigold volatiles affects proximal potato plants, but stronger defense induction and a more efficient approach should be obtained after prolonged and/or repeated stimuli, which will be in focus of our further investigations. It would be of great interest to study in more detail the effect of this prolonged exposure on, as shown here, affected starch metabolism, especially in terms of starch stored in tubers.

## Figures and Tables

**Table 1:** Composition of French Marigold (*Tagetes patula* L.) essential oil.

	Compound	RI	%
1	*α*-Pinene	932	1.1
2	Camphene	947	0.1
3	Sabinene	972	1.2
4	*β*-Pinene	976	0.3
5	Myrcene	996	0.1
6	ni	998	0.1
7	*α*-Phellandrene	1006	0.3
8	ni	1007	0.2
9	para-Cymene	1024	tr
10	Limonene	1027	14.7
11	(*Z*)-*β*-Ocimene	1038	10.8
12	(*E*)-*β*-Ocimene	1047	0.7
13	dihydro-Tagetone	1051	1.2
14	ni	1054	0.1
15	*γ*-Terpinene	1058	0.1
16	Terpinolene	1090	32.4
17	ni	1132	0.6
18	*E*-Myroxide	1141	0.2
19	*E*-Tagetone	1145	1.5
20	*Z*-Tagetone	1150	3.1
21	ni	1176	1.3
22	*α*-Terpineol	1189	0.2
23	Methyl chavicol	1203	tr
24	Thymol, methyl ether	1234	0.2
25	Piperitone	1252	8.2
26	Bornyl acetate	1284	0.2
27	Dihydroedulan II	1287	0.3
28	ni	1393	0.2
29	*E*-Caryophyllene	1419	12.2
30	*α*-Humulene	1454	0.2
31	Germacrene D	1482	1.6
32	Bicyclogermacrene	1498	1.2
33	*δ*-Cadinene	1525	0.1
34	ni	1554	0.3
35	ni	1558	0.1
36	Spathulenol	1579	1.0
37	Caryophyllene oxide	1583	2.5
38	Caryophylla-4(12),8(13)-dien-5-alpha-ol	1638	0.4
39	*α*-Cadinol	1656	0.1
40	ni	1663	0.1
41	ni	1700	tr
42	2,6,10-trimethyl,14-ethylene-14-pentadecne	1851	0.8
	Number of detected compounds	42	
	Number of identified compounds	32	
Groups of identified compounds (%):			
Monoterpenes		70.0	
Monoterpenoids		6.6	
Sesqiuterpenes		15.3	
Sesqiterpenoids		4.0	
Diterpenes		0.8	
Others		0.3	
% of identified compounds in total essential oil yield		97.0	

RI—retention index; ni—non-identified; tr—in traces.

**Table 2:** Number of differentially expressed (DE) transcripts, DE portion in total number of identified potato sequences and sequences used for DE analysis (%), up- and down-regulated DE transcripts in varied fold change (FC) cut-offs in potato plants exposed to French Marigold EO for 8 h.

FC	log_2_FC	Number of DE Transcripts	% of Identified Potato Sequences (37891)	% of Sequences Used for DE Analysis (29260)	Up- Regulated	Down- Regulated
>2	>1.00	2796	7.38	9.55	1303	1493
>5	>2.32	485	1.28	1.66	237	248
>10	>3.32	181	0.48	0.62	91	90
>50	>5.64	19	0.05	0.06	10	9
>100	>6.64	8	0.02	0.03	3	5

**Table 3:** Transcripts annotated to sot00500: Starch and sucrose metabolism KEGG pathway with microarray obtained expression rates (fold changes—FC and log_2_FC) in potato plants after 8 h of exposure to French Marigold EO.

No	Prim. Acc.	Description	FC	log_2_FC
1	XM_015309112.1	beta-glucosidase 18-like	−5.696	−2.510
2	XM_015307086.1	granule-bound starch synthase	−5.300	−2.406
3	XM_006343700.2	granule-bound starch synthase	−5.091	−2.348
4	XM_015307087.1	granule-bound starch synthase	−5.042	−2.334
5	XM_015307085.1	granule-bound starch synthase	−4.993	−2.320
6	XM_006343701.2	granule-bound starch synthase	−4.979	−2.316
7	XM_015307083.1	granule-bound starch synthase	−4.860	−2.281
8	XM_015307084.1	granule-bound starch synthase	−4.847	−2.277
9	XM_006360516.2	beta-amylase-like	−4.389	−2.134
10	XM_006360533.1	beta-amylase-like	−3.082	−1.624
11	NM_001287886.1	beta-fructofuranosidase	−2.994	−1.582
12	XM_006361941.2	endoglucanase 24-like	−2.910	−1.541
13	XM_006366431.2	beta-glucosidase 13-like	−2.749	−1.459
14	XM_006348168.2	beta-glucosidase 18-like	−2.592	−1.374
15	XM_006340834.2	beta-amylase 1, chloroplastic	−2.579	−1.367
16	NM_001287989.1	granule-bound starch synthase	−2.539	−1.344
17	XM_006354826.2	alpha-amylase-like	−2.274	−1.185
18	XM_006360882.2	probable sucrose-phosphate synthase 2	−2.142	−1.099
19	XM_006343977.2	beta-glucosidase BoGH3B-like	−2.124	−1.087
20	XM_006347657.2	beta-glucosidase BoGH3B	2.022	1.016
21	NM_001288308.1	sucrose synthase	2.035	1.025
22	XM_006353177.2	endoglucanase 12	2.398	1.262
23	XM_015302882.1	beta-glucosidase BoGH3B-like	2.553	1.352
24	XM_015302878.1	beta-glucosidase BoGH3B-like	2.581	1.368
25	XM_015302885.1	beta-glucosidase BoGH3B-like	2.634	1.397
26	XM_006359111.2	furcatin hydrolase	3.038	1.603
27	XM_006353435.2	hexokinase-1	3.218	1.686
28	XM_006353434.2	hexokinase-1	3.254	1.702
29	XM_015311367.1	hexokinase-1	3.320	1.731
30	XM_006357203.2	alpha-amylase 3, chloroplastic	3.354	1.746
31	XM_006347240.2	fructokinase-like	3.458	1.790
32	NM_001288199.1	alpha-1,4 glucanphosphorylase L-2	3.774	1.916
33	XM_006361066.2	beta-glucosidase BoGH3B-like	3.850	1.945
34	XM_006361064.2	beta-glucosidase BoGH3B-like	3.959	1.985
35	NM_001288357.1	Sucrose synthase (SS16)	4.469	2.160
36	XM_006350146.2	beta-glucosidase BoGH3B-like	6.630	2.729
37	XM_006353704.2	sucrose synthase 2	9.560	3.257
38	XM_015311444.1	sucrose synthase 2	9.626	3.267
39	NM_001287982.1	sucrose synthase 2	9.795	3.292

**Table 4:** Soluble sugars (fructose, glucose and sucrose) content (µg mL^−1^) in control potato plants (C) and after 8 h of exposure to French Marigold EO. Ratios of 8 h/C values were presented as color-coded heat map (blue-decreased ratio; white-unchanged; red-increased ratio).

	C	8 h	log_2_ (8 h/C)	
Fructose	7.35 ± 2.80	5.60 ± 1.03	−0.39	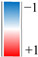
Glucose	40.41 ± 19.26	21.30 ± 7.83	−0.92	
Sucrose	0.76 ± 0.26	1.17 ± 0.46	0.62	

All data were statistically compared by Student’s *t*-test (*p* < 5 × 10^−2^). Results were presented as means ± SE (n = 3).

## Supplementary Materials

Supplementary materials can be found at https://www.mdpi.com/2223-7747/10/1/172/s1, Table S1: Sequences of primers used in RT-qPCR analyses. Table S2: List of DE transcripts associated to GO, Interpro and KEGG annotations.
