# Supplementary material for: Transcriptome Profiling of the Potato Exposed to French Marigold Essential Oil with a Special Emphasis on Leaf Starch Metabolism and Defense against Colorado Potato Beetle
Source: Plants (Basel). 2021 Jan 18;10(1):172. doi: 10.3390/plants10010172 (PMC7831324; doi:10.3390/plants10010172)
Supplement: Supplementary file 1 [file plants-10-00172-s001.zip › Supplementary material/Table S1.docx]

| **Gene** | **Protein** | **GenBank™**  **Accession Number** | **Primer sequence ( 5’- 3’)** | **Sequence**  **length (bp)** |  |
| --- | --- | --- | --- | --- | --- |
| *PGMP* | Phosphoglucomutase, chloroplastic | NM_001288352.1 | F:AGCTGCAGCACCTAGAGTTT  R:TCACCAACAGCCAGTACGAG | 131 | |
| *AGPS1* | Glucose-1-phosphate adenylyltransferase | XM_006365058.2 | F:GTTGACTCCCCGAAGGAAGC  R:ACATGAACTAGGCAAAGTGCAAC | 105 | |
| *GBSS* | Granule-bound starch synthase, chloroplastic | XM_015307086.1 | F:CAAAATGATTGGTTTTTGCTGGGG  R:TCTATTGACTTTGATCCACACAACC | 110 | |
| *SS1* | Starch synthase 1 | NM_001288145.1 | F:CTGGGAAAATGCAGCCATTCA  R:AATAGCACCAAAAGCTCCGC | 137 | |
| *GWD* | Alpha-glucan water dikinase 1, chloroplastic | NM_001288123.1 | F:ACGTGATCCAAAGCCATCACA  R:ATTTCTCCCTATATCATACCCAGC | 134 | |
| *DPE* | 4-alpha-glucanotransferase | NM_001287852.1 | F:CACAGTTCACCTCATTGCTGC  R:GGAAAGGCTCTAGCATTCGT | 155 |  |
| *PGLCT* | Hexose transporter | XM_015313620.1 | F:GAAGATGATCTGCGTATGATTGAG  R:TATTATCATCTTGCACACCTACGG | 111 |  |
| *AMY3* | Alpha-amylase 3, chloroplastic | XM_006357203.2 | F:GCCTTTTCAGAAGATAAAGAGGCA  R:CTGAACCAGCAACTCAATTGTTTA | 169 |  |
| *MEX1* | Maltose excess protein 1, chloroplastic | XM_006356232.2 | F:GATTTGGTGAAAGTGCTTTTGGC  R:CATGTAAAAGAACCACAGGAACC | 110 |  |
| *BAM* | Beta-amylase 1, chloroplastic | XM_006340834.2 | F:TGCACTAAGCAAATGGTTGTC  R:CATATAAGAAGAACGATAGCGAGC | 114 |  |
| *18S* | 18S ribosomal RNA | X16077.1 | F:TGACGGAGAATTAGGGTTCG  R:CAATGGATCCTCGTTAAGGG | 190 |  |

**Table S1.** Sequences of primers used in RT-qPCR analyses
